# Giant hydatid cyst of the liver with a retroperitoneal growth: a case report

**DOI:** 10.1186/1752-1947-6-298

**Published:** 2012-09-13

**Authors:** Giuseppe Maria Ettorre, Giovanni Vennarecci, Roberto Santoro, Andrea Laurenzi, Cecilia Ceribelli, Antonio Di Cintio, Elisa Busi Rizzi, Mario Antonini

**Affiliations:** 1 Division of Surgical Oncology and Liver Transplantation, San Camillo Hospital, POIT San Camillo-INMI Lazzaro Spallanzani, Cir.ne Gianicolense N° 187, 00100, Rome, Italy; 2 Department of Radiology, INMI Lazzaro Spallanzani, POIT San Camillo-INMI Lazzaro Spallanzani, Rome, Italy; 3 Intensive Care Unit, INMI Lazzaro Spallanzani, POIT San Camillo-INMI Lazzaro Spallanzani, Rome, Italy

## Abstract

**Introduction:**

Hydatid disease is a helminthic anthropozoonosis with worldwide distribution due to the close associations among sheep, dogs, and humans. It can occur almost anywhere in the body with a variety of imaging features, which may change according to the growth stage, associated complications, and affected tissues. A definitive diagnosis requires a combination of imaging, serologic and immunologic studies. Ultrasonography, computed tomography and magnetic resonance imaging are highly accurate in detecting a hepatic hydatid cyst. However, hepatic hydatid cysts in an unusual location and/or of an unusual dimension, with atypical imaging findings, may complicate the differential diagnosis. Surgical treatment remains the best treatment.

**Case presentation:**

We describe an unusual case of a giant hydatid cyst, with exophytic growth from the right lobe of the liver of a 55-year-old Egyptian man. The cyst was strongly adhered to his ipsilateral kidney, which was displaced in a downwards and anterior direction, close to his abdominal wall, simulating a retroperitoneal origin. This atypical growth raised doubts about the most appropriate surgical approach. Magnetic resonance imaging easily clarified the origin of the cyst as our patient’s liver, allowing accurate surgical planning.

**Conclusion:**

Rarely, hydatid cysts can reach an extremely large size without any additional symptoms. Giant cysts need radical therapy because they might lead to perforation and anaphylaxis in some patients. Magnetic resonance imaging is very useful in the study of hydatid disease because of its capacity to allow a large field of view, multiplanar acquisition, and high contrast resolution. In some unusual hepatic presentations, magnetic resonance imaging can be used to determine the correct anatomical relationships.

## Introduction

Hydatid disease (HD) is a helminthic anthropozoonosis with worldwide distribution, caused by the larval stage of the *Echinococcus* tapeworm. It can occur almost anywhere in the body with a variety of imaging features, which may change according to the growth stage, associated complications and affected tissues.

A definitive diagnosis requires a combination of imaging, serologic and immunologic studies. Hepatic hydatid cysts can be treated by surgery, chemotherapy and/or percutaneous aspiration. Although a number of scolecoidal agents have been developed against HD, surgical resection is the only curative treatment. HD primarily affects the liver and demonstrates characteristic imaging findings on ultrasonography (US); computed tomography (CT) and magnetic resonance imaging (MRI) are highly accurate in detecting hepatic HD.

However, hepatic hydatid cysts in an unusual location or of an unusual dimension, with atypical imaging findings, may complicate the differential diagnosis. Large cysts, today fairly rare even in endemic areas, are called giant hydatid cysts (GHCs) [1,2]. GHCs have exophytic growth through the natural routes provided by the liver capsule, ligaments and peritoneum. In these cases, MRI is the most useful tool among the imaging techniques to demonstrate cyst migration.

We describe an unusual case of a GHC originating from the right lobe of the liver with exophytic growth into the retroperitoneum. This atypical growth raised doubts about the most appropriate surgical approach. MRI easily clarified the origin of the cyst as the liver, allowing accurate surgical planning.

## Case presentation

A 55-year-old Egyptian man reported to our department with a large cystic mass occupying his entire right abdominal quadrants, detected by abdominal US during a routine check-up. The features of the mass on US were consistent with HD and it was diagnosed as three cysts - hepatic, renal and retroperitoneal. Our patient had no history of liver or kidney disease or pain in his right abdominal quadrants. Serologic and immunologic studies (indirect hemagglutination, enzyme immunoassays) performed during his hospitalization confirmed the HD diagnosis and a surgical approach was the first choice because of the large dimension of the mass. However, on the basis of US images alone, it was difficult to understand the origin of the cysts and to determine if there were three cysts or just a single giant cyst.

**Figure 1:**
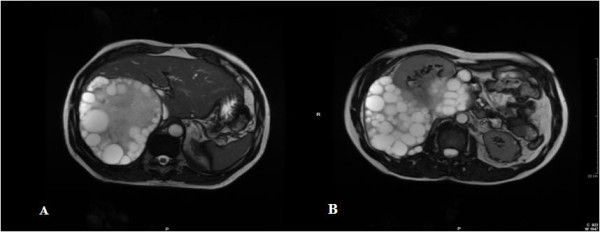
**Magnetic resonance imaging showing a huge single 30**×**18**×**16cm cystic multiloculated mass.** (**A**, **B**) Axial steady state sequence. The cyst displaced the right kidney, simulating the growth of a retroperitoneal mass.

**Figure 2:**
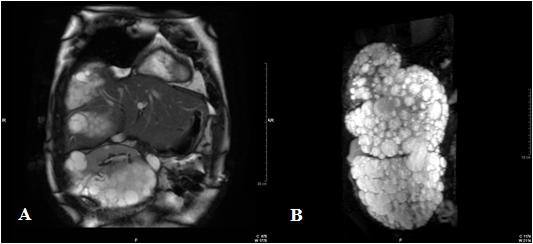
(A) Axial steady state sequence shows the cyst adherent to the right hepatic lobe and to the right kidney which is displaced into the pelvis, (B) Three-dimensional T2-weighted sequence shows the giant multiloculated liver cyst with its exophytic growth.

Our patient underwent unenhanced MRI to determine preoperatively the extent of the disease. The MRI scans showed a huge single 30×18×16cm cystic multiloculated mass, misdiagnosed as three single cysts, hepatic and renal, during the US investigation (Figures 1 and 2). The cyst was strongly adhered to his right hepatic lobe and to his right kidney. The intestinal loops were shifted to the left and his kidney was rotated and displaced down and anteriorly with partial compression of his inferior vena cava.

**Figure 3:**
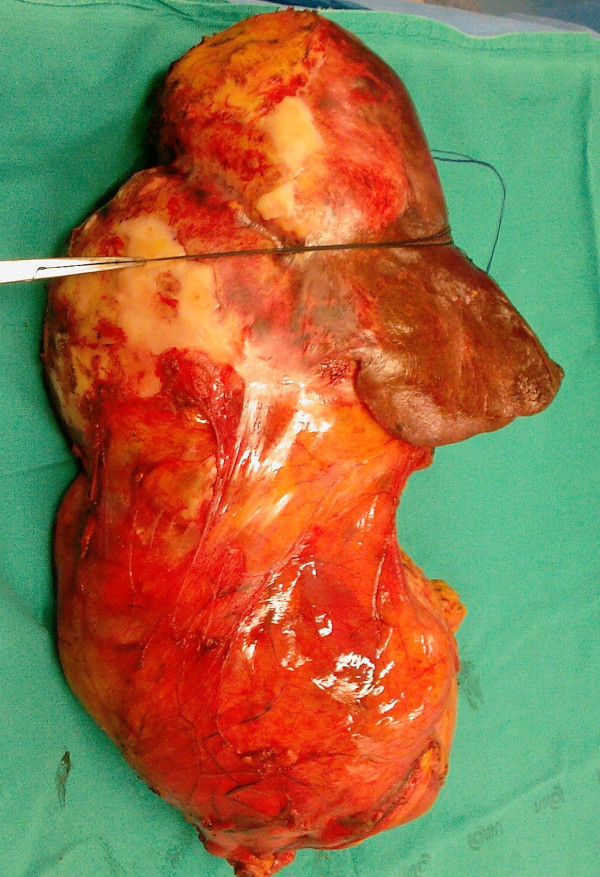
**Specimen of** ***en bloc*** **resection of right liver and giant hydatid cyst.**

Our patient was then scheduled for surgery. A week before surgery, oral therapy with albendazole was started and continued until one month after surgery. A laparotomy was performed via a bisubcostal incision with extension to the xiphoid. The mass was freed from its adhesions with his stomach and small bowel. His inferior vena cava, suprahepatic region and the space between his right and middle hepatic veins was dissected to perform the Belghiti liver hanging maneuver [3]. A tape was then passed between his liver and the anterior surface of his inferior vena cava. We performed a right hepatectomy, with associated *en bloc* hydatid mass resection with an anterior approach, leaving his liver with the mass in place during hepatic transection (Figure 3). Intermittent clamping of the hepatic pedicle was used during transection to minimize bleeding from the cut surface of his liver. His postoperative course was uneventful and our patient was discharged on the seventh day after the surgery.

## Discussion

Echinococcal cysts are mostly found in the liver (75%) and lungs (15%); less frequently they involve anatomical locations such as the brain, bones and heart [4].

The clinical manifestations of hepatic HD depend on the site, size and stage of development of the cyst [5] and HD is largely asymptomatic until complications occur [6]. Uncomplicated liver cysts are always initially asymptomatic, especially when they are small, well-encapsulated or calcified cysts. Rupture of the cyst because of trauma or hyper-pressure from the growing cyst are the most common complications, and may cause anaphylactic shock or the formation of secondary echinococcosis [4].

It has been reported that hepatic hydatid cysts grow one centimeter in diameter during the first six months and then two to three centimeters annually; this is strictly related to the surrounding tissue resistance [7]. GHCs are extremely rare and, in advanced disease, complications are common [8,9].

Typical radiological findings are well known and very helpful in the diagnosis of the disease. US, CT and MRI are highly accurate in detecting hepatic HD [10,11], and several classification schemes based on cyst appearance have been proposed [1,12]. US is the first diagnostic technique for hepatic HD and no further imaging techniques are requested when the appearance is typical [13].

CT and MRI may display the same findings as US; however, calcification of the cyst wall or internal septa is easily detected with CT. It is widely accepted that abdominal CT, with its higher rate of accuracy, is the diagnostic tool of choice. However, MRI, due to its multiplanar capabilities and the excellent contrast resolution for soft tissues, has a particular importance if the diagnosis of HD is questionable, because it is more accurate in demonstrating parietal features and defining anatomical relationships.

Furthermore, hepatic HD may have a low-signal intensity rim on T2-weighted MR images, which probably represents the outer layer (pericyst), rich in collagen and generated by the host. It has been proposed as a characteristic but nonspecific sign [10] when comparing these lesions with nonparasitic epithelial cysts.

The treatment options for hydatid cyst of the liver depend on stage, localization, size and complications of the cysts, and include nonoperative and operative methods [14]. Operative methods include classical surgical techniques (total or subtotal cyst-pericystectomy, partial hepatectomy, capsulorrhaphy, capitonnage, omentoplasty); minimally invasive techniques, such as laparoscopic or robotic procedures; and other treatment modalities, such as puncture, aspiration, injection and reaspiration of scolicidal solutions. When used alone, chemotherapy with antihelmintics of the benzimidazole family has limited efficacy, mostly related to the accessibility of the cyst to the drug. This treatment outcome is better when used as an adjunct to surgery to prevent recurrence [15]. Indications for surgery are the following: active cyst, complicated cyst (infection, compression and obstruction), cyst located near vital organs (central nervous system, spinal cord and heart) and giant cyst at risk of rupture in pleural and/or abdominal cavities. Radical surgical resection remains the mainstay of curative treatment and an accurate preoperative localization is essential [16,17]. In our experience, regulated liver resections (major hepatectomy or segmental resection) instead of total or subtotal cyst-pericystectomy are preferable because of a reduced risk of disease recurrence, intraoperative contamination and postoperative complications (biliary fistula, abdominal collection and bleeding). As a consequence, patients with giant hepatic HD should be referred to high volume hepatobiliary centers. Furthermore, it is important to highlight that GHCs usually do not infiltrate adjacent organs but have strong inflammatory adhesions, which allows the surgeon to avoid multiorgan resections.

In our case, the large dimension and proximity to vital organs induced us to operate so as to avoid cystic complications, such as a rupture in the pleural cavity through the diaphragm or inferior vena cava compression. Moreover, the challenge of this case lay in the conspicuity of the HD. Indeed, the hepatic cyst reported here was an extremely rare large asymptomatic cyst originating from the right lobe of our patient’s liver, was well-encapsulated, was not calcified, had a unique retroperitoneal growth, and was strongly adhered to his right kidney, peritoneum and diaphragm, thus suggesting a retroperitoneal origin of the mass.

Hepatic HD may use two common routes of exophytic growth: the bare area of the liver and the gastrohepatic ligament. Growth through the bare area leads to involvement of the diaphragm and extension into the thorax; growth through the gastrohepatic ligament appears to be the path by which the cyst reaches the stomach. In our case, the giant size of the mass created doubt about its origin - hepatic or retroperitoneal. This was because the cyst rotated and displaced his right kidney down and anteriorly, simulating the growth of a retroperitoneal mass.

In this case, multiplanar MRI was the best imaging technique to demonstrate exophytic cyst growth and its migration toward the retroperitoneum and diaphragm, and allowed accurate presurgical diagnosis and surgical planning (that is, the surgical approach: anterior/bisubcostal laparotomy, lombotomy thoracophrenolaparotomy). For these reasons, we believe that MRI is mandatory in the case of a complex hydatid cyst.

## Conclusion

We report that a hydatid cyst can, rarely, reach an extremely large size without any additional symptoms, and that these giant cysts need radical therapy because they might lead to perforation and anaphylaxis in some patients.

MRI is very useful in the study of HD, because of its capacity to allow a large field of view, multiplanar acquisition and high contrast resolution. In some unusual hepatic presentations, MRI can be used to determine the correct anatomical relationships. Finally, radical surgical resection remains the best treatment option.

## Consent

Written informed consent was obtained from our patient for the publication of this case report and any accompanying images. A copy of the written consent is available for review by the Editor-in-Chief of this journal.

## Abbreviations

CT: computed tomography; GHC: giant hydatid cysts; HD: hydatid disease; MRI: magnetic resonance imaging; US: ultrasonography.

## Competing interests

The authors declare that they have no competing interests.

## Authors’ contributions

GME and GV wrote the paper and performed the surgery. AL, CC, RS, PL, EBR, MA and ADC collected data, and performed the MRI imaging and surgery. All authors read and approved the final manuscript.
